# Supplementary material for: Knowledge synthesis of 100 million biomedical documents augments the deep expression profiling of coronavirus receptors
Source: eLife. 2020 May 28;9:e58040. doi: 10.7554/eLife.58040 (PMC7371427; doi:10.7554/eLife.58040)
Supplement: Supplementary file 1. — The set of studies which have currently been analyzed and made accessible for analysis in the Single Cell Platform are listed below. [file elife-58040-supp1.docx]

**Supplementary File 1. List of studies included in the Single Cell Platform**

The set of studies which have currently been analyzed and made accessible for analysis in the Single Cell Platform are listed below.

| Nference  ID | StudyTitle | Journal | Reference | Species | Technology | ACE2  expression | Considered for analysis |
| --- | --- | --- | --- | --- | --- | --- | --- |
| 1 | A single-cell survey of the small intestinal epithelium | Nature | PMID: 29144463  [(Haber et al., 2017)](https://paperpile.com/c/Hasaq7/M5VJ) | Mus musculus | 10X | Yes | Yes |
| 10 | Mature Kidney - Spatiotemporal immune zonation of the human kidney | Science | PMID: 31604275  [(Stewart et al., 2019)](https://paperpile.com/c/Hasaq7/f45d) | Homo sapiens | 10X | Yes | Yes |
| 11 | Identification of grade and origin specific cell populations in serous epithelial ovarian cancer by single cell RNA-seq | PLoS One | PMID: 30383866  [(Shih et al., 2018)](https://paperpile.com/c/Hasaq7/71NJ) | Homo sapiens | 10X | Yes | Yes |
| 12 | A human liver cell atlas reveals heterogeneity and epithelial progenitors. | Nature | PMID:31292543  [(Aizarani et al., 2019)](https://paperpile.com/c/Hasaq7/0K8J) | Homo sapiens | CELSeq2 | Yes | Yes |
| 13 | Human Pancreas scRNA-seq (Integration of 3 Datasets) | Cell Stem Cell | PMID:27345837  [(Grün et al., 2016)](https://paperpile.com/c/Hasaq7/n23y) | Homo sapiens | CelSeq | Yes | Yes |
| 13 | Human Pancreas scRNA-seq (Integration of 3 Datasets) | Cell Metabolism | PMID:27667667  [(Segerstolpe et al., 2016)](https://paperpile.com/c/Hasaq7/Ul43) | Homo sapiens | SmartSeq2 | Yes | Yes |
| 13 | Human Pancreas scRNA-seq (Integration of 3 Datasets) | Cell Systems | PMID:27693023  [(Muraro et al., 2016)](https://paperpile.com/c/Hasaq7/7Vxa) | Homo sapiens | CelSeq2 | Yes | Yes |
| 14 | Census Of Immune Cells | HCA - Single Cell Portal | <https://data.humancellatlas.org/explore/projects/cc95ff89-2e68-4a08-a234-480eca21ce79> | Homo sapiens | 10X_V2 | No | Yes |
| 15 | Mapping the Mouse Cell Atlas by Microwell-Seq. | Cell | PMID:29474909  [(Han et al., 2018)](https://paperpile.com/c/Hasaq7/0MBY) | Mus musculus | MicrowellSeq | Yes | Yes |
| 16 | Transcriptome Landscape of Human Folliculogenesis Reveals Oocyte and Granulosa Cell Interactions. | Molecular Cell | PMID: 30472193  [(Zhang et al., 2018)](https://paperpile.com/c/Hasaq7/KhWR) | Homo sapiens | SmartSeq2 | No | No |
| 17 | A Cellular Anatomy of the Normal Adult Human Prostate and Prostatic Urethra. | Cell Reports | PMID: 30566875  [(Henry et al., 2018)](https://paperpile.com/c/Hasaq7/kRLx) | Homo sapiens | 10X | Yes | Yes |
| 18 | Single-cell reconstruction of the early maternal–fetal interface in humans | Nature | PMID: 30429548  [(Vento-Tormo et al., 2018)](https://paperpile.com/c/Hasaq7/Qest) | Homo sapiens | 10X | Yes | Yes |
| 2 | Single-cell transcriptomics of 20 mouse organs creates a Tabula Muris. | Nature | PMID:30283141  [(Tabula Muris Consortium et al., 2018)](https://paperpile.com/c/Hasaq7/ivLU) | Mus musculus | 10X | Yes | Yes |
| 2 | Single-cell transcriptomics of 20 mouse organs creates a Tabula Muris. | Nature | PMID:30283141  [(Tabula Muris Consortium et al., 2018)](https://paperpile.com/c/Hasaq7/ivLU) | Mus musculus | FACS | Yes | Yes |
| 20 | Single-Cell Transcriptomic Analysis of Primary and Metastatic Tumor Ecosystems in Head and Neck Cancer | Cell | PMID: 29198524  [(Puram et al., 2017)](https://paperpile.com/c/Hasaq7/unz8) | Homo sapiens | SmartSeq2 | Yes | No |
| 21 | Single-cell transcriptomic atlas of the human retina identifies cell types associated with age-related macular degeneration | Nature Communications | PMID: 31653841  [(Menon et al., 2019)](https://paperpile.com/c/Hasaq7/6CDX) | Homo sapiens | 10X | No | Yes |
| 22 | Single-cell reconstruction of the adult human heart during heart failure and recovery reveals the cellular landscape underlying cardiac function | Nature Cell Biology | PMID:31915373  [(L. Wang et al., 2020)](https://paperpile.com/c/Hasaq7/qWeN) | Homo sapiens | 10X | Yes | Yes |
| 23 | Single cell analysis reveals immune cell-adipocyte crosstalk regulating the transcription of thermogenic adipocytes | eLife | PMID: 31644425  [(Rajbhandari et al., 2019)](https://paperpile.com/c/Hasaq7/TL7r) | Mus musculus | Drop-Seq | Yes | Yes |
| 24 | An atlas of the aging lung mapped by single cell transcriptomics and deep tissue proteomics | Nature Communications | PMID: 30814501  [(Angelidis et al., 2019)](https://paperpile.com/c/Hasaq7/HFzB) | Mus musculus | Drop-Seq | Yes | Yes |
| 25 | The adult human testis transcriptional cell atlas | Cell Research | PMID: 30315278  [(Guo et al., 2018)](https://paperpile.com/c/Hasaq7/Dfuo) | Homo sapiens | 10X | Yes | Yes |
| 26 | Single-cell reconstruction of follicular remodeling in the human adult ovary | Nature Communications | PMID: 31320652  [(Fan et al., 2019)](https://paperpile.com/c/Hasaq7/PxMa) | Homo sapiens | 10X | Yes | Yes |
| 27 | Single-cell analysis of olfactory neurogenesis and differentiation in adult humans | Nature Neuroscience | PMID: 32066986  [(Durante et al., 2020)](https://paperpile.com/c/Hasaq7/41CU) | Homo sapiens | 10X | Yes | Yes |
| 28 | Single-Cell Transcriptomic Map of the Human and Mouse Bladders | Journal of the American Society of Nephrology | PMID: 31462402  [(Yu et al., 2019)](https://paperpile.com/c/Hasaq7/IvqY) | Homo sapiens | 10X | Yes | Yes |
| 29 | Single cell analysis reveals immune cell-adipocyte crosstalk regulating the transcription of thermogenic adipocytes | eLife | PMID: 31644425  [(Rajbhandari et al., 2019)](https://paperpile.com/c/Hasaq7/TL7r) | Mus musculus | 10X - Nuc-Seq | Yes | Yes |
| 3 | Intra- and Inter-cellular Rewiring of the Human Colon during Ulcerative Colitis | Cell | PMID:31348891  [(Smillie et al., 2019)](https://paperpile.com/c/Hasaq7/xIze) | Homo sapiens | 10X | Yes | Yes |
| 30 | Single-cell analysis of human adipose tissue identifies depot- and disease-specific cell types | Nature Metabolism | PMID: 32066997  [(Vijay et al., 2020)](https://paperpile.com/c/Hasaq7/0zce) | Homo sapiens | 10X | Yes | Yes |
| 31 | Adipose tissue - Construction of a human cell landscape at single-cell level | Nature | <https://www.nature.com/articles/s41586-020-2157-4>  32214235  [(Han et al., 2020)](https://paperpile.com/c/Hasaq7/eUpq) | Homo sapiens | Microwell-Seq | Yes | Yes |
| 32 | Adrenal gland - Construction of a human cell landscape at single-cell level | Nature | <https://www.nature.com/articles/s41586-020-2157-4>  32214235  [(Han et al., 2020)](https://paperpile.com/c/Hasaq7/eUpq) | Homo sapiens | Microwell-Seq | Yes | Yes |
| 33 | Artery - Construction of a human cell landscape at single-cell level | Nature | <https://www.nature.com/articles/s41586-020-2157-4>  32214235  [(Han et al., 2020)](https://paperpile.com/c/Hasaq7/eUpq) | Homo sapiens | Microwell-Seq | Yes | Yes |
| 34 | Ascending colon - Construction of a human cell landscape at single-cell level | Nature | <https://www.nature.com/articles/s41586-020-2157-4>  32214235  [(Han et al., 2020)](https://paperpile.com/c/Hasaq7/eUpq) | Homo sapiens | Microwell-Seq | Yes | Yes |
| 35 | Bladder - Construction of a human cell landscape at single-cell level | Nature | <https://www.nature.com/articles/s41586-020-2157-4>  32214235  [(Han et al., 2020)](https://paperpile.com/c/Hasaq7/eUpq) | Homo sapiens | Microwell-Seq | Yes | Yes |
| 36 | Bone marrow - Construction of a human cell landscape at single-cell level | Nature | <https://www.nature.com/articles/s41586-020-2157-4>  32214235  [(Han et al., 2020)](https://paperpile.com/c/Hasaq7/eUpq) | Homo sapiens | Microwell-Seq | No | Yes |
| 37 | Cerebellum - Construction of a human cell landscape at single-cell level | Nature | <https://www.nature.com/articles/s41586-020-2157-4>  32214235  [(Han et al., 2020)](https://paperpile.com/c/Hasaq7/eUpq) | Homo sapiens | Microwell-Seq | No | Yes |
| 38 | Cervix - Construction of a human cell landscape at single-cell level | Nature | <https://www.nature.com/articles/s41586-020-2157-4>  32214235  [(Han et al., 2020)](https://paperpile.com/c/Hasaq7/eUpq) | Homo sapiens | Microwell-Seq | No | Yes |
| 39 | Small intestine duodenum - Construction of a human cell landscape at single-cell level | Nature | <https://www.nature.com/articles/s41586-020-2157-4>  32214235  [(Han et al., 2020)](https://paperpile.com/c/Hasaq7/eUpq) | Homo sapiens | Microwell-Seq | Yes | Yes |
| 4 | Immune Cell Atlas: Blood Mononuclear Cells (2 donors, 2 sites) | HCA - Single Cell Portal | <https://singlecell.broadinstitute.org/single_cell/study/SCP345/ica-blood-mononuclear-cells-2-donors-2-sites> | Homo sapiens | 10X | Yes | Yes |
| 40 | Appendix - Construction of a human cell landscape at single-cell level | Nature | <https://www.nature.com/articles/s41586-020-2157-4>  32214235  [(Han et al., 2020)](https://paperpile.com/c/Hasaq7/eUpq) | Homo sapiens | Microwell-Seq | Yes | Yes |
| 41 | Esophagus - Construction of a human cell landscape at single-cell level | Nature | <https://www.nature.com/articles/s41586-020-2157-4>  32214235  [(Han et al., 2020)](https://paperpile.com/c/Hasaq7/eUpq) | Homo sapiens | Microwell-Seq | Yes | Yes |
| 42 | Fallopian tube - Construction of a human cell landscape at single-cell level | Nature | <https://www.nature.com/articles/s41586-020-2157-4>  32214235  [(Han et al., 2020)](https://paperpile.com/c/Hasaq7/eUpq) | Homo sapiens | Microwell-Seq | Yes | Yes |
| 43 | Gallbladder - Construction of a human cell landscape at single-cell level | Nature | <https://www.nature.com/articles/s41586-020-2157-4>  32214235  [(Han et al., 2020)](https://paperpile.com/c/Hasaq7/eUpq) | Homo sapiens | Microwell-Seq | Yes | Yes |
| 44 | Heart - Construction of a human cell landscape at single-cell level | Nature | <https://www.nature.com/articles/s41586-020-2157-4>  32214235  [(Han et al., 2020)](https://paperpile.com/c/Hasaq7/eUpq) | Homo sapiens | Microwell-Seq | Yes | Yes |
| 45 | Small intestine ileum - Construction of a human cell landscape at single-cell level | Nature | <https://www.nature.com/articles/s41586-020-2157-4>  32214235  [(Han et al., 2020)](https://paperpile.com/c/Hasaq7/eUpq) | Homo sapiens | Microwell-Seq | Yes | Yes |
| 46 | Small intestine jejunum - Construction of a human cell landscape at single-cell level | Nature | <https://www.nature.com/articles/s41586-020-2157-4>  32214235  [(Han et al., 2020)](https://paperpile.com/c/Hasaq7/eUpq) | Homo sapiens | Microwell-Seq | Yes | Yes |
| 47 | Kidney - Construction of a human cell landscape at single-cell level | Nature | <https://www.nature.com/articles/s41586-020-2157-4>  32214235  [(Han et al., 2020)](https://paperpile.com/c/Hasaq7/eUpq) | Homo sapiens | Microwell-Seq | Yes | Yes |
| 48 | Liver - Construction of a human cell landscape at single-cell level | Nature | <https://www.nature.com/articles/s41586-020-2157-4>  32214235  [(Han et al., 2020)](https://paperpile.com/c/Hasaq7/eUpq) | Homo sapiens | Microwell-Seq | Yes | Yes |
| 49 | Lung - Construction of a human cell landscape at single-cell level | Nature | <https://www.nature.com/articles/s41586-020-2157-4>  32214235  [(Han et al., 2020)](https://paperpile.com/c/Hasaq7/eUpq) | Homo sapiens | Microwell-Seq | Yes | Yes |
| 5 | Spleen - Ischaemic sensitivity of human tissue by single cell RNA seq | Human Cell Atlas | <https://data.humancellatlas.org/explore/projects/c4077b3c-5c98-4d26-a614-246d12c2e5d7> | Homo sapiens | 10X_V2 | Yes | Yes |
| 50 | Muscle - Construction of a human cell landscape at single-cell level | Nature | <https://www.nature.com/articles/s41586-020-2157-4>  32214235  [(Han et al., 2020)](https://paperpile.com/c/Hasaq7/eUpq) | Homo sapiens | Microwell-Seq | Yes | Yes |
| 51 | Omental adipose tissue - Construction of a human cell landscape at single-cell level | Nature | <https://www.nature.com/articles/s41586-020-2157-4>  32214235  [(Han et al., 2020)](https://paperpile.com/c/Hasaq7/eUpq) | Homo sapiens | Microwell-Seq | Yes | Yes |
| 52 | Pancreas - Construction of a human cell landscape at single-cell level | Nature | <https://www.nature.com/articles/s41586-020-2157-4>  32214235  [(Han et al., 2020)](https://paperpile.com/c/Hasaq7/eUpq) | Homo sapiens | Microwell-Seq | Yes | Yes |
| 53 | Peripheral blood - Construction of a human cell landscape at single-cell level | Nature | <https://www.nature.com/articles/s41586-020-2157-4>  32214235  [(Han et al., 2020)](https://paperpile.com/c/Hasaq7/eUpq) | Homo sapiens | Microwell-Seq | No | Yes |
| 54 | Lung pleura - Construction of a human cell landscape at single-cell level | Nature | <https://www.nature.com/articles/s41586-020-2157-4>  32214235  [(Han et al., 2020)](https://paperpile.com/c/Hasaq7/eUpq) | Homo sapiens | Microwell-Seq | Yes | Yes |
| 55 | Prostate - Construction of a human cell landscape at single-cell level | Nature | <https://www.nature.com/articles/s41586-020-2157-4>  32214235  [(Han et al., 2020)](https://paperpile.com/c/Hasaq7/eUpq) | Homo sapiens | Microwell-Seq | Yes | Yes |
| 56 | Rectum - Construction of a human cell landscape at single-cell level | Nature | <https://www.nature.com/articles/s41586-020-2157-4>  32214235  [(Han et al., 2020)](https://paperpile.com/c/Hasaq7/eUpq) | Homo sapiens | Microwell-Seq | Yes | Yes |
| 57 | Sigmoid colon - Construction of a human cell landscape at single-cell level | Nature | <https://www.nature.com/articles/s41586-020-2157-4>  32214235  [(Han et al., 2020)](https://paperpile.com/c/Hasaq7/eUpq) | Homo sapiens | Microwell-Seq | Yes | Yes |
| 58 | Spleen - Construction of a human cell landscape at single-cell level | Nature | <https://www.nature.com/articles/s41586-020-2157-4>  32214235  [(Han et al., 2020)](https://paperpile.com/c/Hasaq7/eUpq) | Homo sapiens | Microwell-Seq | No | Yes |
| 59 | Stomach - Construction of a human cell landscape at single-cell level | Nature | <https://www.nature.com/articles/s41586-020-2157-4>  32214235  [(Han et al., 2020)](https://paperpile.com/c/Hasaq7/eUpq) | Homo sapiens | Microwell-Seq | No | Yes |
| 6 | Esophagus - Ischaemic sensitivity of human tissue by single cell RNA seq | Human Cell Atlas | <https://data.humancellatlas.org/explore/projects/c4077b3c-5c98-4d26-a614-246d12c2e5d7>  32214235  [(Han et al., 2020)](https://paperpile.com/c/Hasaq7/eUpq) | Homo sapiens | 10X_V2 | Yes | Yes |
| 60 | Brain temporal lobe - Construction of a human cell landscape at single-cell level | Nature | <https://www.nature.com/articles/s41586-020-2157-4>  32214235  [(Han et al., 2020)](https://paperpile.com/c/Hasaq7/eUpq) | Homo sapiens | Microwell-Seq | Yes | Yes |
| 61 | Thyroid - Construction of a human cell landscape at single-cell level | Nature | <https://www.nature.com/articles/s41586-020-2157-4>  32214235  [(Han et al., 2020)](https://paperpile.com/c/Hasaq7/eUpq) | Homo sapiens | Microwell-Seq | Yes | Yes |
| 62 | Trachea - Construction of a human cell landscape at single-cell level | Nature | <https://www.nature.com/articles/s41586-020-2157-4>  32214235  [(Han et al., 2020)](https://paperpile.com/c/Hasaq7/eUpq) | Homo sapiens | Microwell-Seq | Yes | Yes |
| 63 | Transverse colon - Construction of a human cell landscape at single-cell level | Nature | <https://www.nature.com/articles/s41586-020-2157-4>  32214235  [(Han et al., 2020)](https://paperpile.com/c/Hasaq7/eUpq) | Homo sapiens | Microwell-Seq | Yes | Yes |
| 64 | Ureter - Construction of a human cell landscape at single-cell level | Nature | <https://www.nature.com/articles/s41586-020-2157-4>  32214235  [(Han et al., 2020)](https://paperpile.com/c/Hasaq7/eUpq) | Homo sapiens | Microwell-Seq | No | Yes |
| 65 | Uterus - Construction of a human cell landscape at single-cell level | Nature | <https://www.nature.com/articles/s41586-020-2157-4>  32214235  [(Han et al., 2020)](https://paperpile.com/c/Hasaq7/eUpq) | Homo sapiens | Microwell-Seq | Yes | Yes |
| 8 | A revised airway epithelial hierarchy includes CFTR-expressing ionocytes | Nature | PMID: 30069044  [(Montoro et al., 2018)](https://paperpile.com/c/Hasaq7/ZFIT) | Mus musculus | 10X | Yes | Yes |
| 9 | Fetal Kidney - Spatiotemporal immune zonation of the human kidney | Science | PMID: 31604275  [(Stewart et al., 2019)](https://paperpile.com/c/Hasaq7/f45d) | Homo sapiens | 10X | Yes | Yes |
| 19 | Single-cell transcriptome analysis reveals differential nutrient absorption functions in human intestine | JEM | PMID: 31753849  [(Y. Wang et al., 2020)](https://paperpile.com/c/Hasaq7/cWkW) | Homo sapiens | 10X | Yes | Yes |
| 66 | SARS-CoV-2 receptor ACE2 and TMPRSS2 are predominantly expressed in a transient secretory cell type in subsegmental bronchial branches | EMBO J | PMID: 32246845  [(Lukassen et al., 2020)](https://paperpile.com/c/Hasaq7/DRmz) | Homo sapiens | 10X | Yes | Yes |
| 67 | SARS-CoV-2 receptor ACE2 and TMPRSS2 are predominantly expressed in a transient secretory cell type in subsegmental bronchial branches | bioRxiv | PMID: 32246845  [(Lukassen et al., 2020)](https://paperpile.com/c/Hasaq7/DRmz) | Homo sapiens | 10X | Yes | Yes |
| 68 | Lung - scRNA-seq assessment of the human lung, spleen, and esophagus tissue stability after cold preservation | Genome Biology | PMID: 31892341  [(Madissoon et al., 2019)](https://paperpile.com/c/Hasaq7/Co8g) | Homo sapiens | 10X | Yes | Yes |
| 69 | Esophagus - scRNA-seq assessment of the human lung, spleen, and esophagus tissue stability after cold preservation | Genome Biology | PMID: 31892341  [(Madissoon et al., 2019)](https://paperpile.com/c/Hasaq7/Co8g) | Homo sapiens | 10X | Yes | Yes |
| 7 | Lung - A cellular census of human lungs identifies novel cell states in health and in asthma | Nature Medicine | PMID: 31209336  [(Vieira Braga et al., 2019)](https://paperpile.com/c/Hasaq7/Cane) | Homo sapiens | DropSeq | Yes | Yes |
| 71 | A single-cell atlas of the human healthy airways | BioRxiv | <https://www.sciencedirect.com/science/article/pii/S0092871420305671>  [(Deprez et al., n.d.)](https://paperpile.com/c/Hasaq7/aMyc) | Homo sapiens | 10X | Yes | Yes |
| 72 | A Single-Cell Transcriptomic Map of the Human and Mouse Pancreas Reveals Inter- and Intra-cell Population Structure | Cell Systems | PMID: 27667365  [(Baron et al., 2016)](https://paperpile.com/c/Hasaq7/OaKm) | Homo sapiens | 10X | Yes | Yes |
| 73 | Transcriptional Programming of Normal and Inflamed Human Epidermis at Single-Cell Resolution | Cell Reports | PMID: 30355494  [(Cheng et al., 2018)](https://paperpile.com/c/Hasaq7/28mV) | Homo sapiens | 10X | Yes | Yes |
| 74 | Massively parallel single-nucleus RNA-seq with DroNc-seq | Nature Methods | PMID: 28846088  [(Habib et al., 2017)](https://paperpile.com/c/Hasaq7/fPj6) | Homo sapiens | Dronc-Seq | No | Yes |
| 75 | Distinct microbial and immune niches of the human colon | Nature Immunology | PMID: 32066951  [(James et al., 2020)](https://paperpile.com/c/Hasaq7/Tl5i) | Homo sapiens | 10X | Yes | Yes |
| 76 | Single cell RNA sequencing of human liver reveals distinct intrahepatic macrophage populations. | Nature Communications | PMID: 30348985  [(MacParland et al., 2018)](https://paperpile.com/c/Hasaq7/JqLn) | Homo sapiens | 10X | Yes | Yes |
| 77 | Single-Cell Analysis of Crohn's Disease Lesions Identifies a Pathogenic Cellular Module Associated with Resistance to Anti-TNF Therapy | Cell | PMID: 31474370  [(Martin et al., 2019)](https://paperpile.com/c/Hasaq7/Shp3) | Homo sapiens | 10X | Yes | Yes |
| 79 | Decoding human fetal liver haematopoiesis. | Nature | PMID: 31597962  [(Popescu et al., 2019)](https://paperpile.com/c/Hasaq7/eAz7) | Homo sapiens | 10X | Yes | Yes |
| 80 | Single-cell transcriptomics of the human retinal pigment epithelium and choroid in health and macular degeneration. | PNAS | PMID: 31712411  [(Voigt et al., 2019)](https://paperpile.com/c/Hasaq7/SQCx) | Homo sapiens | 10X | Yes | Yes |
| 84 | Resolving the fibrotic niche of human liver cirrhosis at single-cell level | Nature | PMID: 31597160  [(Ramachandran et al., 2019)](https://paperpile.com/c/Hasaq7/Nm7f) | Homo sapiens | 10X | Yes | Yes |
| 85 | Nasal cavity - A cellular census of human lungs identifies novel cell states in health and in asthma | Nature Medicine | PMID: 31209336  [(Vieira Braga et al., 2019)](https://paperpile.com/c/Hasaq7/Cane) | Homo sapiens | 10X | Yes | Yes |
| 86 | Virus-inclusive single-cell RNA sequencing reveals the molecular signature of progression to severe dengue | PNAS | PMID: 30530648  [(Zanini et al., 2018)](https://paperpile.com/c/Hasaq7/n3Vw) | Homo sapiens | 10X | Yes | Yes |
| 87 | Host-viral infection maps reveal signatures of severe COVID-19 patients | Cell | 0[(Bost et al., 2020)](https://paperpile.com/c/Hasaq7/0pyG)5687 | Homo sapiens | 10X | No | Yes |
| 88 | SARS-CoV-2 Receptor ACE2 Is an Interferon-Stimulated Gene in Human Airway Epithelial Cells and Is Detected in Specific Cell Subsets across Tissues | Cell | [(Ziegler et al., 2020)](https://paperpile.com/c/Hasaq7/LE3L)PMID: 32413319 | Homo sapiens | Seq-Well | Yes | Yes |
| 89 | SARS-CoV-2 Receptor ACE2 Is an Interferon Stimulated Gene in Human Airway Epithelial Cells and Is Detected in Specific Cell Subsets across Tissues | Cell | [(Ziegler et al., 2020)](https://paperpile.com/c/Hasaq7/LE3L)PM  ID: 32413319 | Macaca mulatta | Seq-Well | Yes | Yes |

[Aizarani N, Saviano A, Sagar, Mailly L, Durand S, Herman JS, Pessaux P, Baumert TF, Grün D. 2019. A human liver cell atlas reveals heterogeneity and epithelial progenitors. *Nature* **572**:199–204.](http://paperpile.com/b/Hasaq7/0K8J)

[Angelidis I, Simon LM, Fernandez IE, Strunz M, Mayr CH, Greiffo FR, Tsitsiridis G, Ansari M, Graf E, Strom T-M, Nagendran M, Desai T, Eickelberg O, Mann M, Theis FJ, Schiller HB. 2019. An atlas of the aging lung mapped by single cell transcriptomics and deep tissue proteomics. *Nat Commun* **10**:963.](http://paperpile.com/b/Hasaq7/HFzB)

[Baron M, Veres A, Wolock SL, Faust AL, Gaujoux R, Vetere A, Ryu JH, Wagner BK, Shen-Orr SS, Klein AM, Melton DA, Yanai I. 2016. A Single-Cell Transcriptomic Map of the Human and Mouse Pancreas Reveals Inter- and Intra-cell Population Structure. *Cell Syst* **3**:346–360.e4.](http://paperpile.com/b/Hasaq7/OaKm)

[Bost P, Giladi A, Liu Y, Bendjelal Y, Xu G, David E, Blecher-Gonen R, Cohen M, Medaglia C, Li H, Deczkowska A, Zhang S, Schwikowski B, Zhang Z, Amit I. 2020. Host-viral infection maps reveal signatures of severe COVID-19 patients. *Cell*. doi:](http://paperpile.com/b/Hasaq7/0pyG)[10.1016/j.cell.2020.05.006](http://dx.doi.org/10.1016/j.cell.2020.05.006)

[Cheng JB, Sedgewick AJ, Finnegan AI, Harirchian P, Lee J, Kwon S, Fassett MS, Golovato J, Gray M, Ghadially R, Liao W, Perez White BE, Mauro TM, Mully T, Kim EA, Sbitany H, Neuhaus IM, Grekin RC, Yu SS, Gray JW, Purdom E, Paus R, Vaske CJ, Benz SC, Song JS, Cho RJ. 2018. Transcriptional Programming of Normal and Inflamed Human Epidermis at Single-Cell Resolution. *Cell Rep* **25**:871–883.](http://paperpile.com/b/Hasaq7/28mV)

[Deprez M, Zaragosi L-E, Truchi M, Garcia SR, Arguel M-J, Lebrigand K, Paquet A, Pee’r D, Marquette C-H, Leroy S, Barbry P. n.d. A single-cell atlas of the human healthy airways. doi:](http://paperpile.com/b/Hasaq7/aMyc)[10.1101/2019.12.21.884759](http://dx.doi.org/10.1101/2019.12.21.884759)

[Durante MA, Kurtenbach S, Sargi ZB, Harbour JW, Choi R, Kurtenbach S, Goss GM, Matsunami H, Goldstein BJ. 2020. Single-cell analysis of olfactory neurogenesis and differentiation in adult humans. *Nat Neurosci* **23**:323–326.](http://paperpile.com/b/Hasaq7/41CU)

[Fan X, Bialecka M, Moustakas I, Lam E, Torrens-Juaneda V, Borggreven NV, Trouw L, Louwe LA, Pilgram GSK, Mei H, van der Westerlaken L, Chuva de Sousa Lopes SM. 2019. Single-cell reconstruction of follicular remodeling in the human adult ovary. *Nat Commun* **10**:3164.](http://paperpile.com/b/Hasaq7/PxMa)

[Grün D, Muraro MJ, Boisset J-C, Wiebrands K, Lyubimova A, Dharmadhikari G, van den Born M, van Es J, Jansen E, Clevers H, de Koning EJP, van Oudenaarden A. 2016. De Novo Prediction of Stem Cell Identity using Single-Cell Transcriptome Data. *Cell Stem Cell* **19**:266–277.](http://paperpile.com/b/Hasaq7/n23y)

[Guo J, Grow EJ, Mlcochova H, Maher GJ, Lindskog C, Nie X, Guo Y, Takei Y, Yun J, Cai L, Kim R, Carrell DT, Goriely A, Hotaling JM, Cairns BR. 2018. The adult human testis transcriptional cell atlas. *Cell Res* **28**:1141–1157.](http://paperpile.com/b/Hasaq7/Dfuo)

[Haber AL, Biton M, Rogel N, Herbst RH, Shekhar K, Smillie C, Burgin G, Delorey TM, Howitt MR, Katz Y, Tirosh I, Beyaz S, Dionne D, Zhang M, Raychowdhury R, Garrett WS, Rozenblatt-Rosen O, Shi HN, Yilmaz O, Xavier RJ, Regev A. 2017. A single-cell survey of the small intestinal epithelium. *Nature* **551**:333–339.](http://paperpile.com/b/Hasaq7/M5VJ)

[Habib N, Avraham-Davidi I, Basu A, Burks T, Shekhar K, Hofree M, Choudhury SR, Aguet F, Gelfand E, Ardlie K, Weitz DA, Rozenblatt-Rosen O, Zhang F, Regev A. 2017. Massively parallel single-nucleus RNA-seq with DroNc-seq. *Nat Methods* **14**:955–958.](http://paperpile.com/b/Hasaq7/fPj6)

[Han X, Wang R, Zhou Y, Fei L, Sun H, Lai S, Saadatpour A, Zhou Z, Chen H, Ye F, Huang D, Xu Y, Huang W, Jiang M, Jiang X, Mao J, Chen Y, Lu C, Xie J, Fang Q, Wang Y, Yue R, Li T, Huang H, Orkin SH, Yuan G-C, Chen M, Guo G. 2018. Mapping the Mouse Cell Atlas by Microwell-Seq. *Cell* **172**:1091–1107.e17.](http://paperpile.com/b/Hasaq7/0MBY)

[Han X, Zhou Z, Fei L, Sun H, Wang R, Chen Y, Chen H, Wang J, Tang H, Ge W, Zhou Y, Ye F, Jiang M, Wu J, Xiao Y, Jia X, Zhang T, Ma X, Zhang Q, Bai X, Lai S, Yu C, Zhu L, Lin R, Gao Y, Wang M, Wu Y, Zhang J, Zhan R, Zhu S, Hu H, Wang C, Chen M, Huang H, Liang T, Chen J, Wang W, Zhang D, Guo G. 2020. Construction of a human cell landscape at single-cell level. *Nature* **581**:303–309.](http://paperpile.com/b/Hasaq7/eUpq)

[Henry GH, Malewska A, Joseph DB, Malladi VS, Lee J, Torrealba J, Mauck RJ, Gahan JC, Raj GV, Roehrborn CG, Hon GC, MacConmara MP, Reese JC, Hutchinson RC, Vezina CM, Strand DW. 2018. A Cellular Anatomy of the Normal Adult Human Prostate and Prostatic Urethra. *Cell Rep* **25**:3530–3542.e5.](http://paperpile.com/b/Hasaq7/kRLx)

[James KR, Gomes T, Elmentaite R, Kumar N, Gulliver EL, King HW, Stares MD, Bareham BR, Ferdinand JR, Petrova VN, Polański K, Forster SC, Jarvis LB, Suchanek O, Howlett S, James LK, Jones JL, Meyer KB, Clatworthy MR, Saeb-Parsy K, Lawley TD, Teichmann SA. 2020. Distinct microbial and immune niches of the human colon. *Nat Immunol* **21**:343–353.](http://paperpile.com/b/Hasaq7/Tl5i)

[Lukassen S, Chua RL, Trefzer T, Kahn NC, Schneider MA, Muley T, Winter H, Meister M, Veith C, Boots AW, Hennig BP, Kreuter M, Conrad C, Eils R. 2020. SARS-CoV-2 receptor ACE2 and TMPRSS2 are primarily expressed in bronchial transient secretory cells. *EMBO J* **39**:e105114.](http://paperpile.com/b/Hasaq7/DRmz)

[MacParland SA, Liu JC, Ma X-Z, Innes BT, Bartczak AM, Gage BK, Manuel J, Khuu N, Echeverri J, Linares I, Gupta R, Cheng ML, Liu LY, Camat D, Chung SW, Seliga RK, Shao Z, Lee E, Ogawa S, Ogawa M, Wilson MD, Fish JE, Selzner M, Ghanekar A, Grant D, Greig P, Sapisochin G, Selzner N, Winegarden N, Adeyi O, Keller G, Bader GD, McGilvray ID. 2018. Single cell RNA sequencing of human liver reveals distinct intrahepatic macrophage populations. *Nat Commun* **9**:4383.](http://paperpile.com/b/Hasaq7/JqLn)

[Madissoon E, Wilbrey-Clark A, Miragaia RJ, Saeb-Parsy K, Mahbubani KT, Georgakopoulos N, Harding P, Polanski K, Huang N, Nowicki-Osuch K, Fitzgerald RC, Loudon KW, Ferdinand JR, Clatworthy MR, Tsingene A, van Dongen S, Dabrowska M, Patel M, Stubbington MJT, Teichmann SA, Stegle O, Meyer KB. 2019. scRNA-seq assessment of the human lung, spleen, and esophagus tissue stability after cold preservation. *Genome Biol* **21**:1.](http://paperpile.com/b/Hasaq7/Co8g)

[Martin JC, Chang C, Boschetti G, Ungaro R, Giri M, Grout JA, Gettler K, Chuang L-S, Nayar S, Greenstein AJ, Dubinsky M, Walker L, Leader A, Fine JS, Whitehurst CE, Mbow ML, Kugathasan S, Denson LA, Hyams JS, Friedman JR, Desai PT, Ko HM, Laface I, Akturk G, Schadt EE, Salmon H, Gnjatic S, Rahman AH, Merad M, Cho JH, Kenigsberg E. 2019. Single-Cell Analysis of Crohn’s Disease Lesions Identifies a Pathogenic Cellular Module Associated with Resistance to Anti-TNF Therapy. *Cell* **178**:1493–1508.e20.](http://paperpile.com/b/Hasaq7/Shp3)

[Menon M, Mohammadi S, Davila-Velderrain J, Goods BA, Cadwell TD, Xing Y, Stemmer-Rachamimov A, Shalek AK, Love JC, Kellis M, Hafler BP. 2019. Single-cell transcriptomic atlas of the human retina identifies cell types associated with age-related macular degeneration. *Nat Commun* **10**:4902.](http://paperpile.com/b/Hasaq7/6CDX)

[Montoro DT, Haber AL, Biton M, Vinarsky V, Lin B, Birket SE, Yuan F, Chen S, Leung HM, Villoria J, Rogel N, Burgin G, Tsankov AM, Waghray A, Slyper M, Waldman J, Nguyen L, Dionne D, Rozenblatt-Rosen O, Tata PR, Mou H, Shivaraju M, Bihler H, Mense M, Tearney GJ, Rowe SM, Engelhardt JF, Regev A, Rajagopal J. 2018. A revised airway epithelial hierarchy includes CFTR-expressing ionocytes. *Nature* **560**:319–324.](http://paperpile.com/b/Hasaq7/ZFIT)

[Muraro MJ, Dharmadhikari G, Grün D, Groen N, Dielen T, Jansen E, van Gurp L, Engelse MA, Carlotti F, de Koning EJP, van Oudenaarden A. 2016. A Single-Cell Transcriptome Atlas of the Human Pancreas. *Cell Syst* **3**:385–394.e3.](http://paperpile.com/b/Hasaq7/7Vxa)

[Popescu D-M, Botting RA, Stephenson E, Green K, Webb S, Jardine L, Calderbank EF, Polanski K, Goh I, Efremova M, Acres M, Maunder D, Vegh P, Gitton Y, Park J-E, Vento-Tormo R, Miao Z, Dixon D, Rowell R, McDonald D, Fletcher J, Poyner E, Reynolds G, Mather M, Moldovan C, Mamanova L, Greig F, Young MD, Meyer KB, Lisgo S, Bacardit J, Fuller A, Millar B, Innes B, Lindsay S, Stubbington MJT, Kowalczyk MS, Li B, Ashenberg O, Tabaka M, Dionne D, Tickle TL, Slyper M, Rozenblatt-Rosen O, Filby A, Carey P, Villani A-C, Roy A, Regev A, Chédotal A, Roberts I, Göttgens B, Behjati S, Laurenti E, Teichmann SA, Haniffa M. 2019. Decoding human fetal liver haematopoiesis. *Nature* **574**:365–371.](http://paperpile.com/b/Hasaq7/eAz7)

[Puram SV, Tirosh I, Parikh AS, Patel AP, Yizhak K, Gillespie S, Rodman C, Luo CL, Mroz EA, Emerick KS, Deschler DG, Varvares MA, Mylvaganam R, Rozenblatt-Rosen O, Rocco JW, Faquin WC, Lin DT, Regev A, Bernstein BE. 2017. Single-Cell Transcriptomic Analysis of Primary and Metastatic Tumor Ecosystems in Head and Neck Cancer. *Cell* **171**:1611–1624.e24.](http://paperpile.com/b/Hasaq7/unz8)

[Rajbhandari P, Arneson D, Hart SK, Ahn IS, Diamante G, Santos LC, Zaghari N, Feng A-C, Thomas BJ, Vergnes L, Lee SD, Rajbhandari AK, Reue K, Smale ST, Yang X, Tontonoz P. 2019. Single cell analysis reveals immune cell-adipocyte crosstalk regulating the transcription of thermogenic adipocytes. *Elife* **8**. doi:](http://paperpile.com/b/Hasaq7/TL7r)[10.7554/eLife.49501](http://dx.doi.org/10.7554/eLife.49501)

[Ramachandran P, Dobie R, Wilson-Kanamori JR, Dora EF, Henderson BEP, Luu NT, Portman JR, Matchett KP, Brice M, Marwick JA, Taylor RS, Efremova M, Vento-Tormo R, Carragher NO, Kendall TJ, Fallowfield JA, Harrison EM, Mole DJ, Wigmore SJ, Newsome PN, Weston CJ, Iredale JP, Tacke F, Pollard JW, Ponting CP, Marioni JC, Teichmann SA, Henderson NC. 2019. Resolving the fibrotic niche of human liver cirrhosis at single-cell level. *Nature* **575**:512–518.](http://paperpile.com/b/Hasaq7/Nm7f)

[Segerstolpe Å, Palasantza A, Eliasson P, Andersson E-M, Andréasson A-C, Sun X, Picelli S, Sabirsh A, Clausen M, Bjursell MK, Smith DM, Kasper M, Ämmälä C, Sandberg R. 2016. Single-Cell Transcriptome Profiling of Human Pancreatic Islets in Health and Type 2 Diabetes. *Cell Metab* **24**:593–607.](http://paperpile.com/b/Hasaq7/Ul43)

[Shih AJ, Menzin A, Whyte J, Lovecchio J, Liew A, Khalili H, Bhuiya T, Gregersen PK, Lee AT. 2018. Identification of grade and origin specific cell populations in serous epithelial ovarian cancer by single cell RNA-seq. *PLoS One* **13**:e0206785.](http://paperpile.com/b/Hasaq7/71NJ)

[Smillie CS, Biton M, Ordovas-Montanes J, Sullivan KM, Burgin G, Graham DB, Herbst RH, Rogel N, Slyper M, Waldman J, Sud M, Andrews E, Velonias G, Haber AL, Jagadeesh K, Vickovic S, Yao J, Stevens C, Dionne D, Nguyen LT, Villani A-C, Hofree M, Creasey EA, Huang H, Rozenblatt-Rosen O, Garber JJ, Khalili H, Desch AN, Daly MJ, Ananthakrishnan AN, Shalek AK, Xavier RJ, Regev A. 2019. Intra- and Inter-cellular Rewiring of the Human Colon during Ulcerative Colitis. *Cell* **178**:714–730.e22.](http://paperpile.com/b/Hasaq7/xIze)

[Stewart BJ, Ferdinand JR, Young MD, Mitchell TJ, Loudon KW, Riding AM, Richoz N, Frazer GL, Staniforth JUL, Vieira Braga FA, Botting RA, Popescu D-M, Vento-Tormo R, Stephenson E, Cagan A, Farndon SJ, Polanski K, Efremova M, Green K, Del Castillo Velasco-Herrera M, Guzzo C, Collord G, Mamanova L, Aho T, Armitage JN, Riddick ACP, Mushtaq I, Farrell S, Rampling D, Nicholson J, Filby A, Burge J, Lisgo S, Lindsay S, Bajenoff M, Warren AY, Stewart GD, Sebire N, Coleman N, Haniffa M, Teichmann SA, Behjati S, Clatworthy MR. 2019. Spatiotemporal immune zonation of the human kidney. *Science* **365**:1461–1466.](http://paperpile.com/b/Hasaq7/f45d)

[Tabula Muris Consortium, Overall coordination, Logistical coordination, Organ collection and processing, Library preparation and sequencing, Computational data analysis, Cell type annotation, Writing group, Supplemental text writing group, Principal investigators. 2018. Single-cell transcriptomics of 20 mouse organs creates a Tabula Muris. *Nature* **562**:367–372.](http://paperpile.com/b/Hasaq7/ivLU)

[Vento-Tormo R, Efremova M, Botting RA, Turco MY, Vento-Tormo M, Meyer KB, Park J-E, Stephenson E, Polański K, Goncalves A, Gardner L, Holmqvist S, Henriksson J, Zou A, Sharkey AM, Millar B, Innes B, Wood L, Wilbrey-Clark A, Payne RP, Ivarsson MA, Lisgo S, Filby A, Rowitch DH, Bulmer JN, Wright GJ, Stubbington MJT, Haniffa M, Moffett A, Teichmann SA. 2018. Single-cell reconstruction of the early maternal-fetal interface in humans. *Nature* **563**:347–353.](http://paperpile.com/b/Hasaq7/Qest)

[Vieira Braga FA, Kar G, Berg M, Carpaij OA, Polanski K, Simon LM, Brouwer S, Gomes T, Hesse L, Jiang J, Fasouli ES, Efremova M, Vento-Tormo R, Talavera-López C, Jonker MR, Affleck K, Palit S, Strzelecka PM, Firth HV, Mahbubani KT, Cvejic A, Meyer KB, Saeb-Parsy K, Luinge M, Brandsma C-A, Timens W, Angelidis I, Strunz M, Koppelman GH, van Oosterhout AJ, Schiller HB, Theis FJ, van den Berge M, Nawijn MC, Teichmann SA. 2019. A cellular census of human lungs identifies novel cell states in health and in asthma. *Nat Med* **25**:1153–1163.](http://paperpile.com/b/Hasaq7/Cane)

[Vijay J, Gauthier M-F, Biswell RL, Louiselle DA, Johnston JJ, Cheung WA, Belden B, Pramatarova A, Biertho L, Gibson M, Simon M-M, Djambazian H, Multiple Tissue Human Expression Resource Consortium, Staffa A, Bourque G, Laitinen A, Nystedt J, Vohl M-C, Fraser JD, Pastinen T, Tchernof A, Grundberg E. 2020. Single-cell analysis of human adipose tissue identifies depot and disease specific cell types. *Nat Metab* **2**:97–109.](http://paperpile.com/b/Hasaq7/0zce)

[Voigt AP, Mulfaul K, Mullin NK, Flamme-Wiese MJ, Giacalone JC, Stone EM, Tucker BA, Scheetz TE, Mullins RF. 2019. Single-cell transcriptomics of the human retinal pigment epithelium and choroid in health and macular degeneration. *Proc Natl Acad Sci U S A* **116**:24100–24107.](http://paperpile.com/b/Hasaq7/SQCx)

[Wang L, Yu P, Zhou B, Song J, Li Z, Zhang M, Guo G, Wang Y, Chen X, Han L, Hu S. 2020. Single-cell reconstruction of the adult human heart during heart failure and recovery reveals the cellular landscape underlying cardiac function. *Nat Cell Biol* **22**:108–119.](http://paperpile.com/b/Hasaq7/qWeN)

[Wang Y, Song W, Wang J, Wang T, Xiong X, Qi Z, Fu W, Yang X, Chen Y-G. 2020. Single-cell transcriptome analysis reveals differential nutrient absorption functions in human intestine. *J Exp Med* **217**. doi:](http://paperpile.com/b/Hasaq7/cWkW)[10.1084/jem.20191130](http://dx.doi.org/10.1084/jem.20191130)

[Yu Z, Liao J, Chen Y, Zou C, Zhang H, Cheng J, Liu D, Li T, Zhang Q, Li J, Yang X, Ye Y, Huang Z, Long X, Yang R, Mo Z. 2019. Single-Cell Transcriptomic Map of the Human and Mouse Bladders. *J Am Soc Nephrol* **30**:2159–2176.](http://paperpile.com/b/Hasaq7/IvqY)

[Zanini F, Robinson ML, Croote D, Sahoo MK, Sanz AM, Ortiz-Lasso E, Albornoz LL, Rosso F, Montoya JG, Goo L, Pinsky BA, Quake SR, Einav S. 2018. Virus-inclusive single-cell RNA sequencing reveals the molecular signature of progression to severe dengue. *Proc Natl Acad Sci U S A* **115**:E12363–E12369.](http://paperpile.com/b/Hasaq7/n3Vw)

[Zhang Y, Yan Z, Qin Q, Nisenblat V, Chang H-M, Yu Y, Wang T, Lu C, Yang M, Yang S, Yao Y, Zhu X, Xia X, Dang Y, Ren Y, Yuan P, Li R, Liu P, Guo H, Han J, He H, Zhang K, Wang Y, Wu Y, Li M, Qiao J, Yan J, Yan L. 2018. Transcriptome Landscape of Human Folliculogenesis Reveals Oocyte and Granulosa Cell Interactions. *Mol Cell* **72**:1021–1034.e4.](http://paperpile.com/b/Hasaq7/KhWR)

[Ziegler CGK, Allon SJ, Nyquist SK, Mbano IM, Miao VN, Tzouanas CN, Cao Y, Yousif AS, Bals J, Hauser BM, Feldman J, Muus C, Wadsworth MH 2nd, Kazer SW, Hughes TK, Doran B, Gatter GJ, Vukovic M, Taliaferro F, Mead BE, Guo Z, Wang JP, Gras D, Plaisant M, Ansari M, Angelidis I, Adler H, Sucre JMS, Taylor CJ, Lin B, Waghray A, Mitsialis V, Dwyer DF, Buchheit KM, Boyce JA, Barrett NA, Laidlaw TM, Carroll SL, Colonna L, Tkachev V, Peterson CW, Yu A, Zheng HB, Gideon HP, Winchell CG, Lin PL, Bingle CD, Snapper SB, Kropski JA, Theis FJ, Schiller HB, Zaragosi L-E, Barbry P, Leslie A, Kiem H-P, Flynn JL, Fortune SM, Berger B, Finberg RW, Kean LS, Garber M, Schmidt AG, Lingwood D, Shalek AK, Ordovas-Montanes J, HCA Lung Biological Network. Electronic address: lung-network@humancellatlas.org, HCA Lung Biological Network. 2020. SARS-CoV-2 Receptor ACE2 Is an Interferon-Stimulated Gene in Human Airway Epithelial Cells and Is Detected in Specific Cell Subsets across Tissues. *Cell*. doi:](http://paperpile.com/b/Hasaq7/LE3L)[10.1016/j.cell.2020.04.035](http://dx.doi.org/10.1016/j.cell.2020.04.035)
